# Doing better: eleven ways to improve the integration of sex and gender in health research proposals

**DOI:** 10.1186/s41073-020-00102-2

**Published:** 2020-11-13

**Authors:** Robin Mason

**Affiliations:** 1 Women’s College Research Institute, Women’s College Hospital, Toronto, Canada; 2 Dalla Lana School of Public Health, University of Toronto, Toronto, Canada

**Keywords:** Sex, Gender, Intersectionality, Research methods

## Abstract

**Background:**

Integrating a sex and gender lens is increasingly recognized as important in health research studies. Past failures to adequately consider sex in drug development, for example, led to medications that were metabolized differently, proved harmful, or ineffective, for females. Including both males and females in study populations is important but not sufficient; health, access to healthcare, and treatment provided are also influenced by gender, the socially mediated roles, responsibilities, and behaviors of boys, girls, women and men. Despite understanding the relevance of sex and gender to health research, integrating this lens into study designs can still be challenging. Identified here, are nine opportunities to address sex and gender and thereby strengthen research proposals.

**Methods:**

Ontario investigators were invited to submit a draft of their health research proposal to the Sex and Gender Research Support Service (SGRSS) at Women’s College Hospital in Toronto, Ontario. The service works to build capacity on the integration of sex, gender, and other identity factors, in health research. Using the SAGER Guidelines and the METRICS for the Study of Sex and Gender in Human Participants as guides, proposals were reviewed to enhance their sex and gender considerations. Content analysis of the feedback provided these investigators was subsequently completed.

**Results:**

Nearly 100 hundred study proposals were reviewed and investigators provided with suggestions on how to enhance their proposal. Analyzing the feedback provided across the reviewed studies revealed commonly overlooked opportunities to elevate consideration of sex and gender. These were organized into nine suggestions to mirror the sections of a research proposal.

**Conclusion:**

Health researchers are often challenged on how to integrate a sex and gender lens into their work. Reviews completed across a range of health research studies show there are several commonly overlooked opportunities to do better in this regard. Nine ways to improve the integration of a sex and gender lens in health research proposals have been identified.

## Background

Sex and gender are recognized as important considerations in the design of health research studies. Sex, the biological and physiological features of males and females should always be considered in research study design. Past failures to include sufficient numbers of females in clinical trials resulted in the marketing of medications that were metabolized differently [1], proved harmful [2], or were ineffective [3], in women. However important it is to ensure the inclusion of both males and females in study populations, it is not sufficient; health, (or its absence), access to healthcare, and treatment provided [4] are also influenced by gender. Gender describes the socially assigned or imposed, roles, responsibilities, and behaviors of girls and boys, women and men. An individual assigned female sex at birth who identifies as a woman is described as a cisgender woman.

Integrating a sex and gender lens has been described as ‘better science’. Doing so increases the relevance and applicability of health research findings to a wider swath of the population and ensures research investments achieve maximum benefit [5]. Increasing recognition of this is evident in requirements enacted by funding bodies such as the American National Institutes of Health and the Canadian Institutes of Health Research (CIHR), that applicants explain how sex and/or gender are considered in their study, or provide convincing reasons for why doing so would be inappropriate. With greater consistency, penalties are being applied during peer review to proposals that have omitted or unsuccessfully argued that sex and gender are irrelevant to the study.

However, culture change can be slow. Despite educational initiatives such as those developed by the Canadian Institutes of Health Research (CIHR) [6], and other supports such as guidelines like, Sex and Gender Equity in Research: Rationale for the SAGER guidelines and recommended use [7] and the Essential Metrics for the Integration of Sex and Gender in Studies with Human Participants [5], many struggle with *how* to integrate a sex and gender lens in their research proposals.

## Method

A Sex and Gender Research Support Service was developed at Women’s College Hospital in Toronto, Ontario to increase investigators’ capacity to consider sex, gender, and identity related factors such as age and race, in their research proposals and projects. Educational modules, online materials, and presentations were developed, and individual support in the form of review and feedback on proposals prior to funding submission provided. Over the past 3 years, more than 80 proposals, with dollar values from several thousand to several million dollars, were reviewed and provided with suggestions on how to better integrate a sex and gender lens.

The feedback that was provided was subsequently reviewed and a modified content analysis completed [8] to determine whether additional tools could or should be developed to support health researchers.

## Results

The content analysis revealed that all of the investigators had missed opportunities to integrate a sex and gender lens and that these ‘missed opportunities’, occurred fairly consistently across the diverse studies. (Although to be fair, not all investigators missed every one of these opportunities, few had fully integrated a sex and gender lens.) The missed opportunities resulted in the identification of nine ways to enhance considerations of sex and gender, as well as other intersecting identity factors, in a research proposal. These are presented below.

### Background and literature review

1. Include in the background, what is known about sex and/or gender issues related to prevalence, presentation, symptoms and treatments for the issue or condition of interest. Note gaps in knowledge when appropriate.
2. In the literature review, note whether past studies integrated a sex and gender lens and included equal or proportional representation of males and females, disaggregated data for analysis, and presented findings by sex, irrespective of whether or not differences were found. Comment on the inclusion or absence of sex/gender, gender identity or other relevant factors in the demographic form, recruitment strategies, results or discussion. The concluding sentence of this section can note sex and gender related gaps and strengthen the rationale for the proposed study.

### Goal and objectives

3. Clearly state who is likely to benefit from the study. Will men, women and gender diverse individuals equally benefit? There is tremendous heterogeneity among men and among women, is the study equally important to all populations? Secondary objectives could explore some of these questions.

### Methods

4. Form an Advisory Committee to help guide the project, especially when a specific health issue or population is the focus of interest. Community members and other relevant stakeholders can help ensure study goals, objectives and methods are relevant, appropriate, and can help with knowledge translation activities.
5. Describe the study population by acknowledging sex and gender.
6. Consider stating, “equal numbers of men and women” or “both men and women” or “men, women and gender diverse individuals” will be recruited, instead of “patients will be recruited”. Sample size should be calculated to, at minimum, support sex disaggregated analysis. Ideally, analysis should be conducted separately for men and women.
7. Consider the language used in the demographic form. If sex and gender are relevant, ask about sex assigned at birth (male, female, intersex) and current gender identity (a drop-down menu may be helpful). Ensure language is inclusive of those whose gender identity is fluid or non-binary, and provide culturally and ethnically suitable options. For example, some Indigenous people describe their sexual, gender and/or spiritual identity as “two-spirited”. Avoid using the word, “other” as an open-ended option.
8. Design recruitment strategies to accommodate those with caregiving responsibilities (should you provide childcare?), income challenges (can you pay for parking or travel costs). Be sensitive to culture and stigma associated with identity or health condition in recruitment materials.
9. When using pre-existing datasets, reference whether disaggregation by sex and gender is possible. Note limitations in the dataset when this is not possible. Consider adding a qualitative component when existing data has no reference to gender and other social determinants of health. If this is not feasible, suggest this may be explored in future studies. Indicate that results will report on sex and gender even when no differences are found.

### Dissemination and knowledge translation

10. Note when opportunities to publish and present at conferences presentation will be made available to all investigators. Describe the ways in which results will be tailored to men, women and relevant sub-populations. Provide examples to enhance the knowledge translation section of the proposal.

### Team description

11. Include both men and women on the study team. Ensure that both men and women are identified in the same way, that is, with or without their titles such as Dr. or Professor. Consider whether the descriptions of all team members are roughly comparable in length style and substance.

## Conclusion

Integrating sex and gender into health research studies is recognized as contributing to the generation of findings relevant to a diverse population. Using a sex and gender lens has been recognized as increasing the rigor, reproducibility and application of research to a real-world, diverse population. However, despite the development of a burgeoning literature, online educational modules and other tools, some investigators continue overlooking opportunities to integrate this lens in their study proposals. Reviews completed across a range of health research studies show there are several commonly overlooked opportunities to do better in this regard.. Integrating these suggestions can improve study design, illustrate the investigator’s familiarity with the issues, and hopefully contribute to greater funding success.
